# Melanoma brain metastases have lower T-cell content and microvessel density compared to matched extracranial metastases

**DOI:** 10.1007/s11060-020-03619-0

**Published:** 2020-09-24

**Authors:** Sarah A. Weiss, Christopher Zito, Thuy Tran, Kazuki Heishima, Veronique Neumeister, John McGuire, Adebowale Adeniran, Harriet Kluger, Lucia B. Jilaveanu

**Affiliations:** 1 Department of Medicine (Medical Oncology), Yale University School of Medicine, New Haven, CT USA; 2 Department of Biology, School of Health and Natural Sciences, University of Saint Joseph, West Hartford, CT USA; 3 Department of Pathology, Yale University School of Medicine, New Haven, CT USA; 4 Section of Medical Oncology, Yale University School of Medicine, 333 Cedar St., New Haven, CT 06520 USA; 5 Present Address: Gifu University, Gifu, Japan; 6 Present Address: Akoya Biosciences, Marlborough, MA USA

**Keywords:** Melanoma, Metastases, Brain, PD-L1, Immune markers, Microvessel density

## Abstract

**Background:**

Although melanoma brain metastases (MBM) tend to respond to systemic therapy concordantly with extracranial metastases, little is known about differences in immune cell and vascular content between the brain and other metastatic sites. Here we studied infiltrating immune cell subsets and microvessel density (MVD) in paired intracerebral and extracerebral melanoma metastases.

**Methods:**

Paired intracerebral and extracerebral tumor tissue was obtained from 37 patients with metastatic melanoma who underwent craniotomy between 1997 and 2014. A tissue microarray was constructed to quantify subsets of tumor-infiltrating T-cell, B-cell, and macrophage content, PD-L1 expression, and MVD using quantitative immunofluorescence.

**Results:**

MBM had lower CD3+ (p = 0.01) and CD4+ (p = 0.003) T-cell content, lower MVD (p = 0.006), and a trend for lower CD8+ (p = 0.17) T-cell content compared to matched extracerebral metastases. There were no significant differences in CD20+ B-cell or CD68+ macrophage content, or tumor or stroma PD-L1 expression. Low MVD (p = 0.008) and high CD68+ macrophage density (p = 0.04) in intracerebral metastases were associated with improved 1-year survival from time of first MBM diagnosis.

**Conclusions:**

Although responses to immune-modulating drugs in the body and the brain tend to be concordant, differences were found in MVD and T-cell content between these sites. Studies of these markers should be incorporated into prospective therapeutic clinical trials to determine their prognostic and predictive value.

## Background

Melanoma is the deadliest cutaneous malignancy and the incidence is rising [1]. Approximately 91,000 new cases were diagnosed in the U.S. in 2018. The 5-year survival rate for patients with metastatic melanoma was historically less than 20% [1], however clinical outcomes have improved since the approval of targeted therapies and immune checkpoint inhibitors (ICI) which now form the foundation of treatment [2–5].

A major challenge in the treatment of patients with advanced melanoma is the development of brain metastases, which cause significant morbidity and mortality and occur in up to 40% of patients, with an even higher percentage reported in autopsy series [6, 7]. MBM are typically managed with local therapy including stereotactic radiosurgery (SRS) or surgical resection depending on the size and number of lesions and presence of neurologic deficits. While local therapy is effective in the control of MBM, it does not impact extracranial disease or other areas of the brain and can be associated with neurologic complications including radionecrosis. Both pembrolizumab [8] and combination ipilimumab and nivolumab have clinical activity against asymptomatic, untreated MBM and have emerged as promising systemic therapy approaches for a select group of MBM patients [9–11].

PD-L1 expression and tumor infiltrating lymphocyte (TIL) content have been associated with response to ICI in extracerebral disease [12, 13]. However, intracranial melanoma metastases are rarely available for analysis, and little is known about how they are similar or different to extracerebral metastases. Moreover, MBMs are sometimes hemorrhagic, a quality that is rarely seen in other sites with the exception of bowel metastases. As MBM treatment paradigms are shifting and less surgery is performed, human MBM tissue remains a valuable but dwindling resource to identify biologic properties of MBM that may differ from extracerebral metastases to rationally inform new treatment strategies. Prognostic and predictive tissue-based biomarkers have been particularly difficult to define for melanoma because of limited access to pre- and post-treatment tissue and due to the technical intricacies of assessing multiple variables in the tumor microenvironment in a simultaneous and standardized manner.

We previously characterized T-cell infiltrates and PD-L1 expression in metastatic melanomas from variable anatomic sites [14]. In that study we profiled a subgroup of 40 MBM which had lower T-cell content compared to unmatched extracerebral metastases. Immune infiltrates have been described in both extracerebral melanoma metastases and MBM but little is known about differences in quantity and function respective to their anatomic sites [15–17]. Our purpose was to further validate and explore the differences in subsets of immune cell infiltrates and PD-L1 expression between matched intracerebral and extracerebral melanoma metastases and to characterize the difference in vessel densities, which has not been previously studied.

## Methods

### Patient cohort and tissue microarray (TMA) construction

Collection of patient specimens and clinical data was approved by the Yale University IRB. The cohort consisted of patients with advanced melanoma who underwent resection of a symptomatic MBM for which both intracerebral and extracerebral tissue from the same patient were available for analysis. Tissue was obtained from the Yale University Department of Pathology Archives. The TMA was constructed from paraffin-embedded, formalin-fixed tissue blocks of three cores taken from a representative region of the tumor. Cores measuring 0.6 mm in diameter were spaced 0.8 mm apart on slides and cut into 5-μm sections and placed on glass slides using an adhesive tape transfer system with UV cross-linking, as previously described [18].

### Quantitative immunofluorescence (QIF) measurements

We stained for markers of T-cell subsets (CD3, CD4, CD8, FOXP3), B-cells (CD20), macrophages (CD68), tumor and stroma PD-L1, and MVD (CD34) in intracerebral and extracerebral melanoma metastases. Briefly, slides or control arrays were concomitantly stained. TMAs were heated, deparaffinized, and rehydrated. Slides were boiled and antigen retrieval was performed.

Slides were incubated with rabbit anti-CD3 (1:100, cat#NB600-1441, Novus) simultaneously with mouse anti-CD8 (1:500, cat#M7103, Dako), and with rabbit anti-CD4 (1:100, cat#M3352, SpringBio) simultaneously with mouse anti-CD20 (1:200, cat#M0755, Dako). Alexa 750 (1:100, Invitrogen) was incubated to label the target. Nuclei were stained with DAPI (Invitrogen). Slides were incubated at 4 °C overnight with mouse anti-FOXP3 (1:200, cat#ab20034, Abcam), mouse anti-CD68 (1:400, cat#M0814, Dako), and with anti-PD-L1 (SP142) (1:200, cat#M4420, SpringBio).

Mouse or rabbit S100 and HMB45 antibodies were utilized to distinguish tumor cells. Goat anti-rabbit or anti-mouse horseradish peroxidase-decorated polymer backbone (Envision, Dako) was utilized to amplify the signal, visualized with Cyanine-3- or Cyanine-5-tyramide. For visualization of S100, a secondary goat IgG conjugated to Alexa 546 (Molecular Probes, Inc.) was utilized for FOXP3, CD68, and PD-L1 and to Alexa 750 (Invitrogen) for CD3/CD8 and CD4/CD20. A nuclear mask was created by incubating the slides with 4,6-diamidine-2-phenylindole (DAPI, 1:500, Invitrogen, Carlsbad, CA).

CD34 was used as a surrogate for microvessel density (MVD) and measured using methods previously described [19]. The image capturing protocol and algorithms used for target expression determination other than PD-L1 have been previously described [20].

### Brain metastasis tumor volume quantitation

Pre-resection MRIs were analyzed using 3D Slicer (https://www.slicer.org), a free, open source medical informatics program for advanced image processing capable of 3D modeling and volumetric analysis [21–23]. Images were manually segmented to differentiate tumor from surrounding normal brain using T1-weighted post-gadolinium images. The Fast GrowCut Extension was applied, and a 3D model was created with Laplacian 30 settings. Five patients were excluded from the tumor volume analyses due to the lack of MRI data available corresponding to the patient-matched TMA core specimen.

### Microvessel morphology assessment

Tumor images stained for CD34 were independently assessed for microvessel morphology in a blinded fashion by two unbiased investigators. The architecture, appearance, and pattern of the vessel layout and organization were examined.

### Statistical analysis

JMP version 5.0.1.2 software was used for all analyses (SAS Institute). The Chi Square test and the t-test were used to test relationships between marker expression levels in intracerebral and extracerebral metastatic sites. For statistical analysis, QIF measurements were analyzed as both continuous variables and classified as high or low using the median score for each marker as a cutoff point. Survival time was calculated as the time from first metastases to death or last follow-up and from first MBM diagnosis to death or last follow-up. Patients who died from a cause other than melanoma or those who were lost to follow-up were censored at the last recorded follow-up time. Cox proportional hazards were used to investigate the association between each variable and survival. Survival curves were generated using the Kaplan–Meier method.

## Results

### Clinical characteristics of matched cohort

The 37 patients included in the study underwent craniotomy for metastatic melanoma between 1997 and 2014. Adequate intracerebral and extracerebral tumor tissue was required for each patient for inclusion. Clinical details are summarized in Table 1.

**Table 1:** Clinical characteristics of the matched patient cohort

Clinical characteristics	No. (total n = 37)	%
Gender		
Male	25	(68)
Female	12	(32)
Age at initial melanoma diagnosis		
Range	19–78 years	–
Mean	51 years	–
Primary melanoma anatomic location		
Trunk	15	(41)
Extremities	7	(19)
Head and neck region	6	(16)
Acral	1	(3)
Unknown primary	2	(5)
Unknown	7	(19)
Primary melanoma thickness		
Range	0.7–11.25 mm	–
Mean	3.9 mm	–
Brain as first site of distant metastases		
Yes	18	(49)
No	17	(46)
Unknown	2	(5)
Craniotomy for melanoma brain metastasis		
Yes	37	(100)
No	0	(0)
Radiation therapy		
SRS	16	(43)
WBRT	4	(11)
SRS and WBRT	9	(24)
EBRT	1	(3)
None or unknown	7	(19)
Timing of first radiation (n = 30)		
Before resection	13	(43)
After resection	16	(53)
Unknown	1	(3)
Systemic therapy: adjuvant		
Interferon	12	(32)
Systemic therapy: before MBM resection (n = 19)		
Chemotherapy	8	(42)
High dose IL-2	6	(32)
Ipilimumab	5	(26)
Interferon	4	(21)
Targeted therapy	2	(11)
Biochemotherapy	1	(5)
Isolated limb perfusion	1	(5)
Vaccine	1	(5)
Anti-PD-1	1	(5)
Unknown	1	(5)
Systemic therapy: after MBM resection (n = 14)		
Chemotherapy	8	(57)
High dose IL-2	5	(36)
Ipilimumab	5	(36)
Targeted therapy	5	(5)
Anti-PD-1	1	(7)
Isolated limb perfusion	1	(7)
Timing of first systemic therapy		
Before MBM resection only	16	(43)
After MBM resection only	6	(16)
Before and after MBM resection	9	(24)
No systemic therapy	3	(8)
Unknown	3	(8)
Time from initial melanoma diagnosis to death or last follow-up		
Range	0.7–18.9 years	–
Mean	5.7 years	–
Time from 1st distant metastasis to death or last follow-up		
Range	0.22–9.8 years	–
Mean	1.8 years	–
Time from 1st brain metastasis to death or last follow-up		
Range	0.13–9.8 years	–
Mean	1.2 years	–

### Immunologic profile of MBM compared to matched extracerebral metastases

Three cores taken from representative regions of each tumor were used on the TMA to determine site-specific differences in inflammation between intracerebral and extracerebral melanoma metastases. We studied the T-cell, B-cell, and macrophage content of the immune infiltrates as well as tumor and stroma PD-L1 expression. We measured percent area of fluorescence per histospot for the T-cell, B-cell and macrophage content and used intensity scores for PD-L1. We used the highest immune cell density in the three histospots and the average fluorescence intensity for PD-L1 since inflammatory cell content can vary within a tumor. These methods were adopted from and are in accordance with other similar studies in the literature [14, 24–26].

We first assessed the correlation of densities of each tumor infiltrating immune cell subset and PD-L1 expression between matched intracerebral and extracerebral tumors using the linear Pearson correlation test (Table 2). Associations were found for CD3+ (R = 0.7), CD8+ (R = 0.55), and FOXP3+ (R = 0.3) TIL and for stromal PD-L1+ cells (R = 0.55). Weaker associations were found for tumor cell PD-L1 expression (R = 0.26). There were no correlations between intracerebral and extracerebral metastases for B-cell or macrophage content.

**Table 2:** Correlation of immune cell content in matched extracerebral and intracerebral metastases

Intracerebral metastases	Extracerebral metastases							
	CD3+	CD4+	CD8+	CD20+	CD68+	Tumor PD-L1	Stroma PD-L1	FOXP3+
CD3+	R = 0.7							
CD4+		R = 0.13						
CD8+			R = 0.55					
CD20+				R = 0.03				
CD68+					R = 0.01			
Tumor PD-L1						R = 0.26		
Stroma PD-L1							R = 0.55	
FOXP3+								R = 0.3

**Fig. 1:**
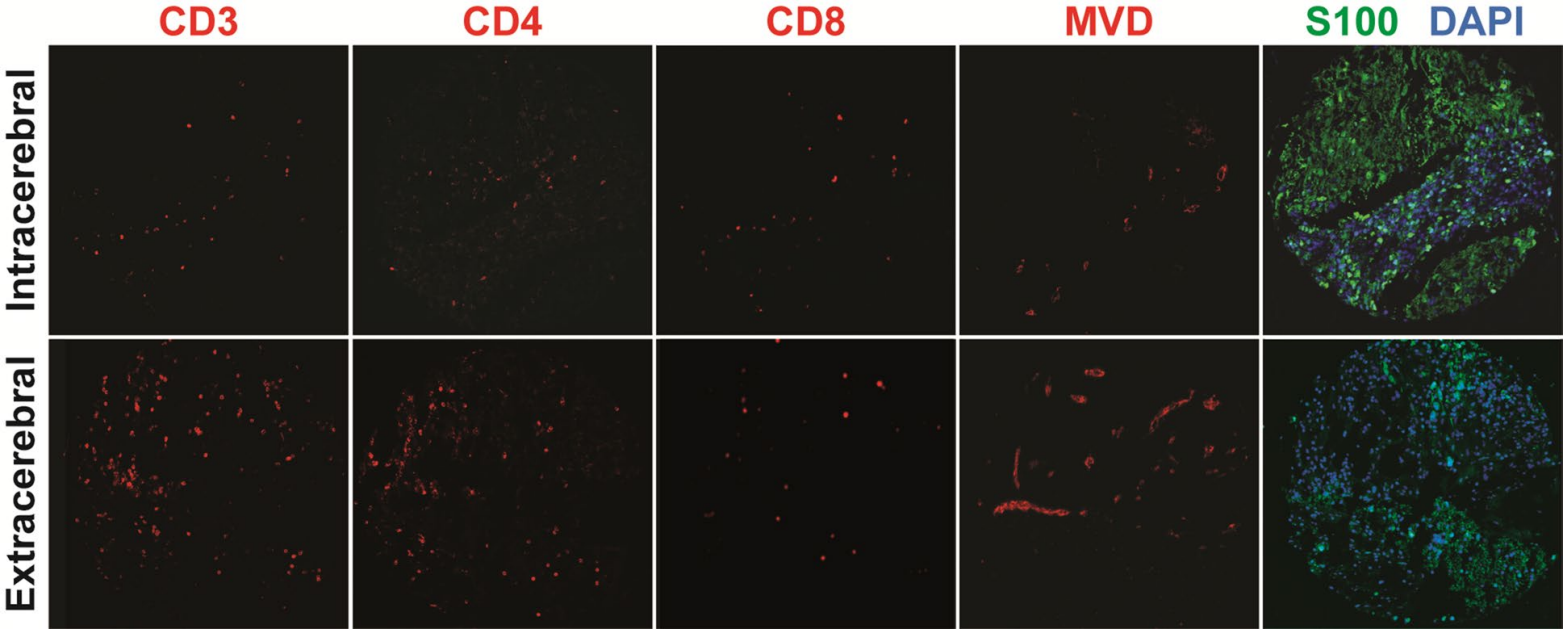
QIF of T-cell content and MVD between paired metastases from a single patient. In a representative patient, quantitative immunofluorescence (QIF) demonstrates lower CD3, CD4, and CD8 T-cell content (red) and lower microvessel density (MVD) (red) in an intracranial metastasis compared to the paired extracranial metastasis. S100 (green) was used to detect tumor cells and a nuclear mask was created by incubating the slides with DAPI (blue)

Next, we analyzed the percent area of each immune cell subset and PD-L1 intensity scores to determine differences in inflammation between intracerebral and extracerebral metastases. Unpaired analysis was performed using the Chi Square test (Table 3). QIF measurements for each marker were designated as high or low using the median value as the cut-off. Extracerebral metastases compared to intracerebral metastases had higher CD3+ (high: 66% vs 35%; p = 0.01), CD4+ (high: 68% vs. 31%; p = 0.003), and lower FOXP3+ TIL content (high: 35% vs. 68%; p = 0.01). The difference in CD8+ content was not statistically significant (p = 0.17). Figure 1 demonstrates QIF of T-cell content between an intracerebral and extracerebral metastasis from the same patient. There were no significant differences in B-cell or macrophage content or tumor or stroma PD-L1 intensity scores between extracerebral and intracerebral metastases. These results were confirmed for CD3 and CD4 using the t-test with QIF measurements for each marker measured as continuous variables. The differences only trended for significance for CD8 and FOXP3 (data not shown). There were no differences in immune cell content or PD-L1 expression in brain metastases when stratified by radiation status.

**Table 3:** Differences in inflammatory cell content between intracerebral and extracerebral metastases

Site	CD3 (p = 0.01)		CD8 (p = 0.17)		CD4 (p = 0.003)		CD20 (p = 0.26)		CD68 (p = 0.32)		Tumor PD-L1 (p = 0.26)		Stroma PD-L1 (p = 0.38)		FOXP3 (p = 0.01)	
	Low	High	Low	High	Low	High	Low	High	Low	High	Low	High	Low	High	Low	High
Brain	21/32	11/32	19/32	13/32	22/32	10/32	20/32	12/32	19/34	15/34	18/32	14/32	18/32	14/32	9/28	19/28
	65.63%	34.38%	59.38%	40.63%	68.75%	31.25%	62.50%	37.50%	55.88%	44.12%	56.25%	43.75%	56.25%	43.75%	32.14%	67.86%
Extra-cerebral	11/32	21/32	13/31	18/31	10/31	21/31	15/31	16/31	14/32	18/32	14/33	19/33	15/33	18/33	20/31	11/31
	34.38%	65.63%	41.94%	58.06%	32.26%	67.74%	48.39%	51.61%	43.75%	56.25%	42.42%	57.58%	45.45%	54.55%	64.52%	35.48%

### Associations between TIL subsets, macrophages, and PD-L1 expression within extracerebral and intracerebral metastases

The Chi Square test was used to analyze associations between inflammatory cell subsets within tumors. Within extracerebral metastases, CD3+, CD4+, CD8+, and CD68+ TIL densities all positively correlated with each other. Stromal PD-L1 intensity correlated with the density of CD3+ (p = 0.03), CD4+ (p = 0.03), CD8+ (p = 0.01), and CD68+ (p = 0.01) cell content. Tumor PD-L1 intensity correlated with stromal PD-L1 (p < 0.0001) and CD4+ (p = 0.01), CD8+ (p = 0.03), and CD68+ (p = 0.02) cell densities as well as with CD3+ TIL, however this association did not reach statistical significance (p = 0.095). Neither FOXP3+ nor CD20+ TIL content correlated with any of the markers.

Similar to extracerebral metastases, CD3+, CD4+, CD8+, and CD68+ cell densities all positively correlated with each other within intracerebral metastases. However, unlike in extracerebral metastases, neither stromal PD-L1 nor tumor PD-L1 expression correlated with CD3+, CD4+, or CD8+ TIL content within intracerebral metastases. Stromal PD-L1 expression had a trend for correlation with CD68+ macrophage content (p = 0.08). Tumor PD-L1 expression correlated with stromal PD-L1 (p = 0.0003) expression and CD68+ (p = 0.01) macrophage content. Neither FOXP3+ nor CD20+ TIL in intracerebral metastases significantly correlated with any of the markers (Supplementary Table 1).

### Microvessel density (MVD) in extracerebral metastases and MBM

**Fig. 2:**
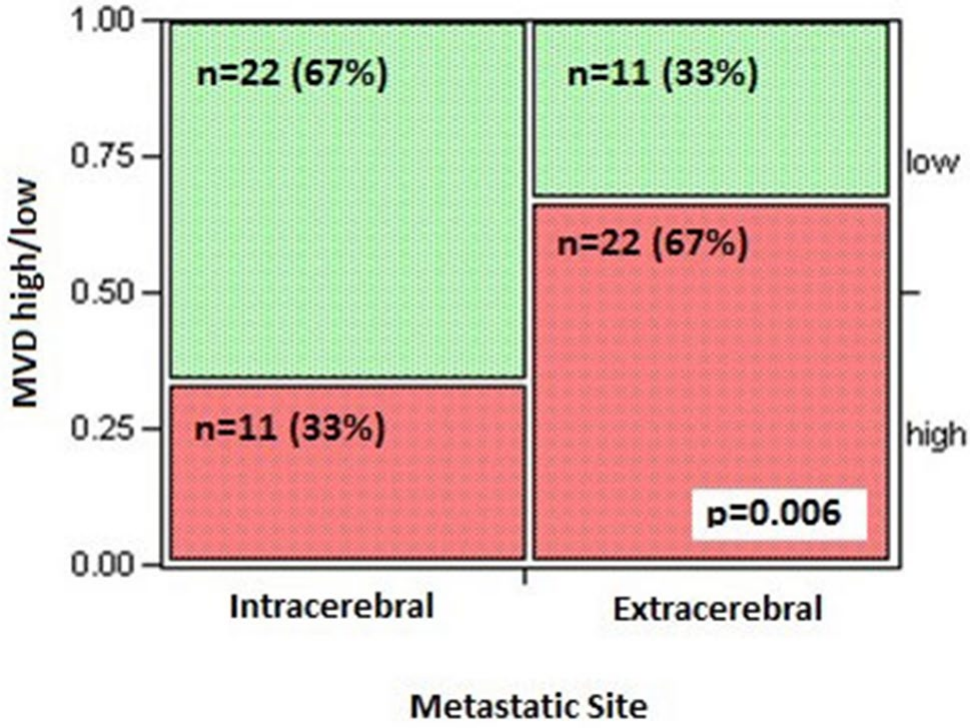
MVD was lower in intracerebral compared to extracerebral metastases. Intracerebral metastases had significantly lower microvessel density (MVD) compared to matched extracerebral metastases by the Chi Square test (p = 0.006)

We next determined site-specific differences in MVD between intracerebral and extracerebral melanoma metastases using anti-CD34 to identify endothelial cells. CD34+ areas were dichotomized into high and low categories by the median score to represent high and low MVD. Intracerebral metastases had significantly lower MVD compared to matched extracerebral metastases by the Chi Square test (p = 0.006, Fig. 2). 22 Out of 33 (67%) MBM had low MVD while only 11/33 (33%) of matched extracerebral tumors had low MVD. MVD level tended to positively correlate between matched intracerebral and extracerebral metastases. MVD level did not significantly correlate with T-cell, B-cell, or macrophage content or with PD-L1 expression in extracerebral or intracerebral metastases (data not shown). There were no significant differences in MVD level in brain metastases when stratified by radiation status. There were no morphologic differences appreciated in the vessels between the brain and extracranial metastatic sites.

### Correlation between clinical characteristics and TIL subsets, macrophages, PD-L1 expression, and MVD

We used the t-test to individually analyze association of age, gender, and primary melanoma tumor thickness with inflammatory cell infiltrates, PD-L1 expression and MVD in both intracerebral and extracerebral metastases.

#### Age

In intracerebral metastases, there was a trend for association between older age and decreased CD3+ (p = 0.07), CD4+ (p = 0.08), and CD8+ (p = 0.07) TIL density, decreased CD68+ (p = 0.09) macrophage content, and lower MVD (p = 0.11). There were no associations of age with any of the markers in extracerebral metastases.

#### Gender

There was no correlation between gender and any of the cell markers in intracerebral metastases. In extracerebral metastases, female sex was associated with increased CD3+ (p = 0.02), CD4+ (p = 0.01), and CD8+ (p = 0.04) TIL densities.

#### Primary tumor thickness

There was a trend for association between thicker primary tumors and decreased CD3+ (p = 0.1) and CD8+ (p = 0.09) T-cell densities and decreased tumor PD-L1 expression in intracerebral metastases. There were no associations between thickness and any of the markers in extracerebral metastases (data not shown).

#### Brain metastasis tumor volume

Brain imaging available on 32 out of 37 patients allowed for calculation of brain metastasis tumor volume. Tumor volume was measured in cubic milliliters, with a range of 0.03 cm^3^ to 36.59 cm^3^. There were no significant associations between brain metastasis tumor volume and TIL subsets, macrophages, PD-L1 expression, or MVD in intracerebral metastases.

### Survival outcomes

**Fig. 3:**
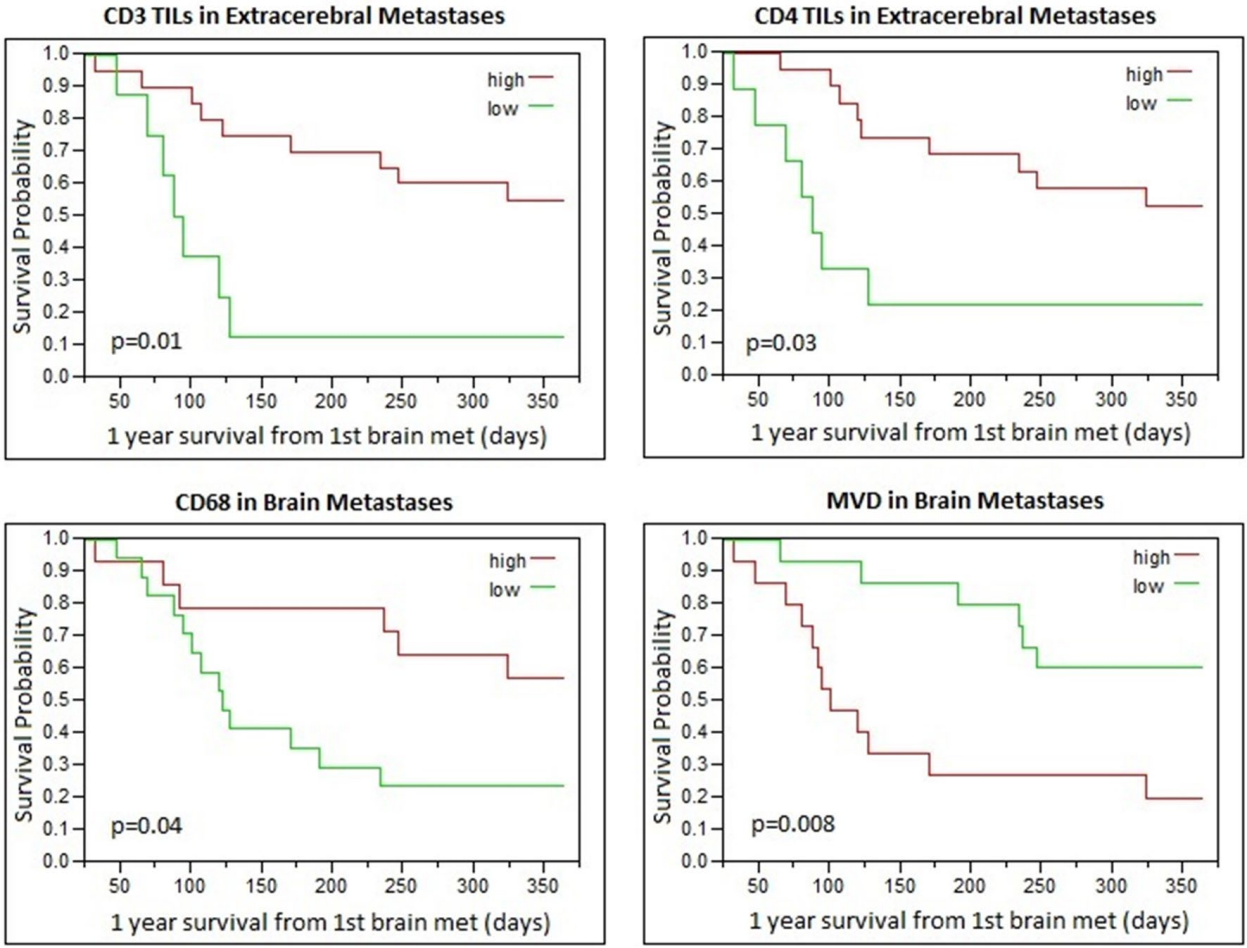
Association of infiltrating immune cell content and MVD with survival from time of MBM diagnosis. In univariate analysis, higher CD3+ and CD4+ T-cell content in extracerebral metastases was associated with improved 1-year survival from the date of first MBM development. In intracerebral metastases, high CD68+ macrophage density and low MVD were associated with improved 1-year survival from the date of first MBM development

In univariate analysis, patients with high extracerebral CD3+ (Cox p = 0.01; RR = 0.5, CI 0.29–0.84) and CD4+ (Cox p = 0.03; RR = 0.6, CI 0.33–0.93) TIL densities had improved 1-year survival from the date of first MBM development. In intracerebral metastases, high CD68+ macrophage density (Cox p = 0.04; RR 0.6, CI 0.36–0.97) and low MVD (Cox p = 0.008; RR = 1.9, CI 1.2–3.3) were associated with improved 1-year survival from the date of first MBM development (Fig. 3).

When controlling for age and gender on multivariate analysis, intracerebral MVD level (p = 0.003) and CD68+ macrophage density (p = 0.06) and extracerebral CD3+ TIL density (p = 0.05) remained associated with 1-year survival from MBM diagnosis. When all three of these markers were incorporated into one multivariate analysis controlling for age and gender, only MVD (p = 0.02) and CD68+ macrophage density (p = 0.02) in MBM retained significance for association with 1-year survival from first MBM development (Supplementary Table 2).

By univariate analysis, we found no difference in survival from time of first distant metastasis among patients whose extracerebral tumors had high versus low T-cell, B-cell or macrophage content nor for PD-L1 expression or MVD level.

## Discussion

We characterized the tumor content of tumor-infiltrating T-cell subsets, B-cells, and macrophages, as well as tumor and stroma PD-L1 expression and MVD in matched extracerebral and intracerebral melanoma metastases to expand upon our prior work in an unmatched cohort [14]. A major strength of this study is the use of matched metastases to allow for intra-patient comparison. The only other studies to our knowledge that examine MBM and extracranial metastases from the same patients include a molecular profiling study in with MBM demonstrating increased expression of proteins in the PI3K/AKT pathway [27] and a whole genome profiling study in which matched MBM and extracranial metastases were more similar to each other than to the primary melanoma [15]. Recently, a RNA sequencing study reported that MBM are characterized by suppression of immune cell networks [17]. Here we confirm our previous findings that MBM have less T-cells compared to extracerebral metastases [16]. MBM had lower CD3+ and CD4+ TIL content and a trend towards lower CD8+ and higher FOXP3+ TIL content compared to matched extracerebral metastases.

Three phase II clinical trials demonstrated clinical activity of the ICI pembrolizumab [11] or ipilimumab plus nivolumab [9, 28] in patients with advanced melanoma and untreated MBM. Although PD-L1 and TIL content are not sufficiently robust at predicting responses to ICI to be clinically useful, understanding the biological differences and similarities between intracerebral and extracerebral metastases within the same patient will enhance development of therapeutic strategies.

The central nervous system (CNS) has previously been described as an immune-privileged site, however several studies have documented lymphocytic infiltration in brain metastases from various cancer types [29, 30]. Immune cell entry into the CNS is highly regulated by complex mechanisms controlling their passage across the blood–brain barrier. Leukocytes can access the brain parenchyma though cerebrospinal fluid via the choroid plexus or directly via blood by attaching to the vascular endothelium and migrating across with the aid of chemokines and other signaling molecules [31]. Despite there being less infiltrating lymphocyte content in MBM, their presence is important. Overcoming immune suppression in the brain will be an important strategy that may augment activity of systemic therapies for MBM. The minimal immune cell density required to impact prognosis or response to immunotherapy is unknown, but clinical trials using immunotherapy in the setting of untreated MBM document concordant responses between MBM and extracranial metastases [8, 9] suggesting these sites do have biologic similarities.

Older age was associated with lower T-cell and macrophage content and lower MVD in brain metastases. Older age has been identified as a negative prognostic factor for survival in MBM patients [32], but studies linking this to decreased immune cell infiltration in MBM are lacking. Low CD68+ macrophage content and high MVD in MBM in our cohort were associated with worse survival from time of MBM diagnosis, independent of age or gender. It may be the case that macrophages in the brain play a key role in immune surveillance and that higher vessel density could provide increased nutrient delivery to MBM thereby promoting their subsistence, but further study is needed.

Another difference we detected was that tumor PD-L1 expression correlated with TILs (CD4, CD8) and macrophages (CD68) in extracerebral sites but not in the brain. Tumor PD-L1 in the brain did correlate with CD68+ macrophage content. PD-L1 expression in tumors often correlates with anti-tumor immune responses, but lymphocyte-rich tumor regions do not always associate with PD-L1 expression. This suggests that TIL presence alone is not sufficient to induce PD-L1 expression [25]. Therefore, PD-L1 expression in MBM in our cohort may be driven by other components of the tumor stroma and microenvironment specific to the brain, such as macrophages. The factors regulating the interactions of microglia and macrophages with the tumor microenvironment in the brain are largely unknown and require further investigation.

Finally, MBM had significantly lower MVD compared to matched extracerebral metastases. Low MVD in MBM was associated with improved survival from the time of first MBM diagnosis. The prognostic value of MVD has previously been examined in melanoma, however the results from these heterogeneous studies are controversial. One study of 45 melanomas from variable sites found that lower central tumor MVD was associated with improved survival [33], however there are no analyses to our knowledge that have characterized MVD in MBM. A meta-analysis also concluded that low tumor MVD in cutaneous melanoma is associated with a higher rate of disease-free survival at up to 60 months [34], however little is known about the importance of MVD in MBM. Unbiased review of vessel morphology between the brain and extracranial metastatic sites did not reveal any appreciable differences, suggesting that the difference in MVD between these sites is not due to morphological changes of the vessels. The size of our cohort is too small to draw conclusions about MVD in MBM without further validation. Future study should include examination of factors in the tumor microenvironment that may impact MVD between anatomic sites. For example, VEGF and ANG2 expression could be quantified and compared between brain and extracranial metastases. Moreover, as drugs targeting VEGF are now being studied in combination with immune therapies (NCT03175432 and NCT02681549), vessel density should be evaluated as a predictive factor for response.

There are several limitations to our study. The sample size is small, yet having matched tissue from the same patient is a rarity and is largely absent from the current literature. The TMA specimens consisted of core biopsies rather than a full excision specimen and thus the possibility of intratumoral heterogeneity may impact the results, although three cores were used for each tumor site. Furthermore, biopsies were not performed at uniform timepoints and the profile of the tumors and their microenvironment can change with disease progression and be affected by systemic therapy and radiation administration. While the sample size was too small to address the impact of individual systemic therapies administered prior to MBM resection on the immune cell content, PD-L1 expression, or MVD, we did demonstrate in this cohort that there were no significant differences in these markers between patients who received radiation therapy prior to MBM resection and those who did not. Additionally, systemic therapy administered after MBM resection in this cohort largely consisted of chemotherapy. Chemotherapy has not been shown to impact survival outcomes in advanced melanoma and therefore the systemic therapy the patients in this cohort received after MBM resection is unlikely to have greatly influenced their survival outcomes. The majority of patients in this study were treated prior to the current era of immune checkpoint inhibitors. For example, only one patient received anti-PD-1 therapy after brain metastasis resection.

Another limitation common to studies like ours is that there are no accepted cut-off points to define QIF positivity for any of the markers we examined. Selecting an arbitrary cut-off point however could lead to an overestimation of differences between the compared groups. Approximately half of metastatic melanomas demonstrate presence of intra-tumor immune infiltrates, and high TIL density correlates with high PD-L1 expression, consistent with findings of Taube et al. and our prior studies [14, 24–26]. Given this reported distribution, we utilized the median value of QIF scores as the threshold for defining “high” versus “low.”

We confirm that MBM have lower T-cell content and we newly report that MBM have lower MVD compared to matched extracerebral melanoma metastases. Despite the lower TIL content in MBM, intracranial response rates to ICI have demonstrated concordance with extracranial responses in clinical trials of patients with untreated MBM [9, 35]. This may suggest that the success of immunotherapy for MBM is less dependent on the T-cell infiltrate and more dependent on other factors, such as degree of immune suppression generated by other cells in the tumor microenvironment, the role of macrophages, or the degree of vessel density that may sustain or stifle MBM growth. Low MVD and high macrophage content in MBM correlated with improved survival from the date of first brain metastasis diagnosis. Better understanding of the immune and vessel composition of extracerebral metastases and its relationship to that of MBM may provide insights that will impact new therapeutic strategies, but larger studies are needed for validation. Tissue-based biomarkers should be incorporated into prospective therapeutic clinical trials to determine their prognostic and/or predictive significance in a larger number of patients under standardized methods.

## Data Availability

The data generated and analyzed in this study are provided in the text and in the figure, tables, and supplementary tables. Any additional data needed can be requested from the corresponding author.

## Abbreviations

CNS: Central nervous system
FDA: Food and Drug Administration
ICI: Immune checkpoint inhibitors
IRB: Institutional Review Board
MBM: Melanoma brain metastases
MVD: Microvessel density
PD-L1: Programmed death ligand 1
QIF: Quantitative immunofluorescence
SRS: Stereotactic radiosurgery
TIL: Tumor infiltrating lymphocytes
TMA: Tissue microarray
WBRT: Whole brain radiation
